# Software that goes with the flow in systems biology

**DOI:** 10.1186/1741-7007-8-140

**Published:** 2010-11-29

**Authors:** Michael Hucka, Nicolas Le Novère

**Affiliations:** 1 Control and Dynamical Systems, California Institute of Technology, Pasadena, CA 91125, USA; 2 EMBL-European Bioinformatics Institute, Wellcome Trust Genome Campus, Hinxton, Cambridgeshire CB10 1SD, UK

## Abstract

**Abstract:**

A recent article in *BMC Bioinformatics *describes new advances in workflow systems for computational modeling in systems biology. Such systems can accelerate, and improve the consistency of, modeling through automation not only at the simulation and results-production stages, but also at the model-generation stage. Their work is a harbinger of the next generation of more powerful software for systems biologists.

See research article: http://www.biomedcentral.com/1471-2105/11/582/abstract/

Ever since the rise of systems biology at the end of the last century, mathematical representations of biological systems and their activities have flourished. They are being used to describe everything from biomolecular networks, such as gene regulation, metabolic processes and signaling pathways, at the lowest biological scales, to tissue growth and differentiation, drug effects, environmental interactions, and more. A very active area in the field has been the development of techniques that facilitate the construction, analysis and dissemination of computational models. The heterogeneous, distributed nature of most data resources today has increased not only the opportunities for, but also the difficulties of, developing software systems to support these tasks. The work by Li *et al*. [1] published in *BMC Bioinformatics *represents a promising evolutionary step forward in this area. They describe a workflow system - a visual software environment enabling a user to create a connected set of operations to be performed sequentially using seperate tools and resources. Their system uses third-party data resources accessible over the Internet to elaborate and parametrize (that is, assign parameter values to) computational models in a semi-automated manner. In Li *et al*.'s work, the authors point towards a promising future for computational modeling and simultaneously highlight some of the difficulties that need to be overcome before we get there.

## Assisting in the creation of computational models

The adoption of standard structured formats for scientific data, such as the Systems Biology Markup Language (SBML) [2], enables software developers to offer a greater diversity of powerful tools to researchers. These tools help to accelerate the pace of research and enable researchers to develop increasingly elaborate theories and models. This trend has been followed not only for the kinds of process-based network models that are SBML's bread and butter, but in other areas of biological research as well (for instance, the PDB format for three-dimensional molecular structures). The past decade has seen the production of a large number of software packages aimed at systems biologists. For example, more than 200 packages are known to support SBML today, embodying a wide variety of capabilities. This abundance of SBML-compatible systems is just one measure of the wealth of software resources available today in systems biology - there exist many other kinds of software resources, such as databases of chemical entities, used routinely by systems biologists.

The more comprehensive modeling environments for systems biology, such as CellDesigner [3], COPASI [4], Virtual Cell [5] and others, have been gradually enriched and now offer a wealth of features for modelers. They provide comprehensive capabilities for working with models once they are created (for example, for parametrization, simulation, analysis and visualization), but as yet, very few widely available systems provide significant automation to assist modelers with the intellectual activity of creating a model in the first place. Some systems, such as CellDesigner and Virtual Cell, now allow the importation of complete ready-to-run models from databases such as BioModels Database [6], which allows researchers to start with an existing base rather than have to recreate everything from scratch. A few others, such as MetNetMaker [7], also provide users with the means to search and import individual reactions and other entities from databases such as the Kyoto Encyclopedia of Genes and Genomes (KEGG) [8], building up a model interactively without having to type in the details of every component. Even fewer systems allow users to search for similar models based on model annotations; one of those few, semanticSBML [9], goes further by providing facilities for clustering models on the basis of similarities in their annotations, and also for merging sets of models into more comprehensive ones.

As capable as these systems are, they still place the responsibility of selecting all the model's components on the modeler's shoulders. Li *et al*.'s efforts push this frontier further forward by allowing a modeler to start with, for example, lists of pathway terms or biochemical entities, and then have the software automatically retrieve matching data about networks, species, and their cellular locations. In the authors' work, this process is guided by a large consensus model of the yeast metabolic network [10]. As more large network maps and models become available to serve as guides, future modelers will increasingly be able to start with existing maps and focus their efforts on creating subsets of the overall system - and that is where automation such as Li *et al*.'s will accelerate research. Their system is not entirely unique in providing this kind of capability (MetNetMaker, mentioned above and which uses the KEGG Ligand database, is another example), but it provides the greatest degree of automation so far, and it offers users the ability to adjust the workflow visually rather than having to rely on fixed procedures that are hard-coded into a given software environment.

## Workflows as sharable data sets in their own right

The procedures required to produce a finished simulation result from a model are important to communicate, publish and store. SBML, by design, only expresses the static structure of a model: the variables and their relationships, and the values of the different numerical constants used in it. SBML does not provide a script for analyzing, simulating or otherwise *doing *something with the model. The nascent Minimum Information About a Simulation Experiment (MIASE) project and its associated effort to develop a structured file format, SED-ML [11], aim to create a software-independent representation of such procedures. A workflow of the sort described in Li *et al*.'s paper is similar to SED-ML but goes farther in both scope and kind. Whereas SED-ML describes the simulation and processing steps that will start with a parametrized model and produce a set of numerical results, Li *et al*.'s workflows start with a qualitative, unparametrized model and apply a wider variety of steps. Starting from a fully annotated, MIRIAM-compliant [12] model, these include, but are not limited to, procedures that can complete the model using a variety of identifier-matching and iterative inference procedures, parametrize the model using experimental data retrieved from multiple online databases, perform structural and numerical validations on the resulting model to help reduce errors, calibrate the model to match more closely some specific experimental conditions, perform parameter optimization via batched distributed processing, generate visualizations of the results, and store numerical results in a software-independent format.

Looking to the future, we believe that, because such workflows can be stored, exchanged and built on (both by humans and by other software workflows), they will eventually become standardized data objects in their own right - stored and exchanged just as models on their own are at the moment. Much as semanticSBML [9] pushes the frontier for comparing sets of SBML-based models, so too, the day will come when researchers perform analyses on sets of workflows. Indeed, Li *et al*. [1] already report an examination of different workflows' execution performance. The possibilities for ingenious new kinds of analyses, transformations, and maybe even automated mutations of workflows provoke the imagination.

## Challenges ahead

Several common problems continue to confront all modelers, including workflow users. The first is simply the lack of fundamental biological data. A frequently cited example are the kinetic data characterizing biochemical reactions. Data resources such as the SABIO-RK (System for the Analysis of Biochemical Pathways - Reaction Kinetics) [13] used by Li *et al*.'s workflow system are making a difference, but the number of biochemical reactions of interest to modelers is vast and the existing data sources are minuscule by comparison. Even more sparse is the information on the locations and amounts of biomolecular participants in cellular reactions. A significant and more fundamental change is also needed in the way the relevant experimental data are produced and shared, in order to keep up with the needs of computational modelers. In particular, the lack of metadata (that is, information about the experimental context used to produce the data, sample information, and post-processing of the experimental results) makes it very hard to evaluate, compare and select suitable data sets.

A second problem is obtaining sufficient annotations from the creators of models so that referenced entities can be uniquely identified and matched to how they are known in centralized databases. Modelers will continue to use their own preferred names for biochemical entities, and that can be perfectly acceptable if they also provide enough identifying information so that those entities can be matched up to appropriate database entries. That is crucial to allowing workflows such as Li *et al*. 's to operate. Contextual information can sometimes be used to disambiguate entities that are poorly specified or identified by uncommon synonyms, but this process is error-prone. The ideal scenario is when the modelers themselves provide sufficient information to uniquely identify what they have in mind. Software tools can help modelers by providing facilities to make the identification process easier, and thankfully some software tools, such as semanticSBML [9], do, but more work is needed in this direction.

A final challenge concerns the long-term survival of software systems and web services. It is all very well to store and exchange workflows, but if the resources they rely upon go out of existence, the workflows become useless. Of course, this challenge is faced by biological research as a whole and is not unique to systems biology. Innovative and useful software packages are continually being created, but they are often small-scale efforts without the means for continued support over years or decades. The loss of these resources wastes time and funding at least twice: the first time when the system is abandoned, and the second time when someone else unknowingly recreates the same thing in a different way. Finding ways to mitigate this problem has been a surprisingly difficult, and so far intractable, challenge.

## References

[B1] Li P Dada JO Jameson D Spasic I Swainston N Carroll K Dunn W Khan F Malys N Messiha HL Simeonidis E Weichart D Winder C Wishart J Broomhead DS Goble CA Gaskell SJ Kell DB Westerhoff HV Mendes P Paton NW Systematic integration of experimental data and models in systems biology BMC Bioinformatics 2010 11 582 10.1186/1471-2105-11-582 PMC3008707 21114840

[B2] Hucka M Finney A Sauro HM Bolouri H Doyle JC Kitano H Arkin AP Bornstein BJ Bray D Cornish-Bowden A Cuellar AA Dronov S Gilles ED Ginkel M Gor V Goryanin II Hedley WJ Hodgman TC Hofmeyr J-HS Hunter PJ Juty NS Kasberger JL Kremling A Kummer U Le Novère N Loew LM Lucio D Mendes P Minch E Mjolsness ED The systems biology markup language (SBML): a medium for representation and exchange of biochemical network models Bioinformatics 2003 19 524 531 10.1093/bioinformatics/btg015 12611808

[B3] Funahashi A Tanimura N Morohashi M Kitano H CellDesigner: a process diagram editor for gene-regulatory and biochemical networks BioSilico 2003 1 159 162 10.1016/S1478-5382(03)02370-9

[B4] Hoops S Sahle S Gauges R Lee C Pahle J Simus N Singhal M Xu L Mendes P Kummer U COPASI - a COmplex PAthway SImulator Bioinformatics 2006 22 3067 3074 10.1093/bioinformatics/btl485 17032683

[B5] Schaff J Fink CC Slepchenko B Carson JH Loew LM A general computational framework for modeling cellular structure and function Biophys J 1997 73 1135 1146 10.1016/S0006-3495(97)78146-3 9284281 PMC1181013

[B6] Li C Donizelli M Rodriguez N Dharuri H Endler L Chelliah V Li L He EU Henry A Stefan MI Snoep JL Hucka M Le Novère N Laibe C BioModels Database: An enhanced, curated and annotated resource for published quantitative kinetic models BMC Syst Biol 2010 4 92 10.1186/1752-0509-4-92 20587024 PMC2909940

[B7] Forth T McConkey GA Westhead DR MetNetMaker: a free and open-source tool for the creation of novel metabolic networks in SBML format Bioinformatics 2010 26 2352 2353 10.1093/bioinformatics/btq425 20671147

[B8] Kanehisa M Goto S KEGG: Kyoto Encyclopedia of Genes and Genomes Nucleic Acids Res 2000 28 27 30 10.1093/nar/28.1.27 10592173 PMC102409

[B9] Krause F Uhlendorf J Lubitz T Schulz M Klipp E Liebermeister W Annotation and merging of SBML models with semanticSBML Bioinformatics 2010 26 421 422 10.1093/bioinformatics/btp642 19933161

[B10] Herrgård MJ Swainston N Dobson P Dunn WB Arga KY Arvas M Blüthgen N Borger S Costenoble R Heinemann M Hucka M Le Novère N Li P Liebermeister W Mo ML Oliveira AP Petranovic D Pettifer S Simeonidis E Smallbone K Spasić I Weichart D Brent R Broomhead DS Westerhoff HV Kırdar B Penttilä M Klipp E Palsson BØ Sauer U Oliver SG A consensus yeast metabolic network reconstruction obtained from a community approach to systems biology Nat Biotechnol 2008 26 1155 1160 10.1038/nbt1492 18846089 PMC4018421

[B11] Köhn D Le Novère N Heiner M, Uhrmacher A SED-ML - an XML format for the implementation of the MIASE guidelines Computational Methods in Systems Biology 2008 Berlin/Heidelberg: Springer 176 190 full_text

[B12] Le Novère N Finney A Hucka M Bhalla US Campagne F Collado-Vides J Crampin E Halstead M Klipp E Mendes P Nielsen P Sauro H Shapiro B Snoep JL Spence HD Wanner BL Minimum Information Requested In the Annotation of biochemical Models (MIRIAM) Nat Biotechnol 2005 23 1509 1515 10.1038/nbt1156 16333295

[B13] Wittig U Golebiewski M Kania R Krebs O Mir S Weidemann A Anstein S Saric J Rojas I Leser U, Naumann F, Eckman B SABIO-RK: integration and curation of reaction kinetics data Data Integration in the Life Sciences 2006 Berlin/Heidelberg, Springer 94 103 full_text

